# Wildlife Monitoring on the Edge: A Performance Evaluation of Embedded Neural Networks on Microcontrollers for Animal Behavior Classification

**DOI:** 10.3390/s21092975

**Published:** 2021-04-23

**Authors:** Juan P. Dominguez-Morales, Lourdes Duran-Lopez, Daniel Gutierrez-Galan, Antonio Rios-Navarro, Alejandro Linares-Barranco, Angel Jimenez-Fernandez

**Affiliations:** 1 Robotics and Tech. of Computers Lab, Universidad de Sevilla, 41012 Seville, Spain; lduran@atc.us.es (L.D.-L.); dgutierrez@atc.us.es (D.G.-G.); arios@atc.us.es (A.R.-N.); alinares@atc.us.es (A.L.-B.); ajimenez@atc.us.es (A.J.-F.); 2 Escuela Técnica Superior de Ingeniería Informática (ETSII), Universidad de Sevilla, 41012 Seville, Spain; 3 Escuela Politécnica Superior, Universidad de Sevilla, 41012 Seville, Spain; 4 Smart Computer Systems Research and Engineering Lab (SCORE), Research Institute of Computer Engineering (I3US), Universidad de Sevilla, 41012 Seville, Spain

**Keywords:** edge-computing, semi-wild animal behavior, neural network, embedded system

## Abstract

Monitoring animals’ behavior living in wild or semi-wild environments is a very interesting subject for biologists who work with them. The difficulty and cost of implanting electronic devices in this kind of animals suggest that these devices must be robust and have low power consumption to increase their battery life as much as possible. Designing a custom smart device that can detect multiple animal behaviors and that meets the mentioned restrictions presents a major challenge that is addressed in this work. We propose an edge-computing solution, which embeds an ANN in a microcontroller that collects data from an IMU sensor to detect three different horse gaits. All the computation is performed in the microcontroller to reduce the amount of data transmitted via wireless radio, since sending information is one of the most power-consuming tasks in this type of devices. Multiples ANNs were implemented and deployed in different microcontroller architectures in order to find the best balance between energy consumption and computing performance. The results show that the embedded networks obtain up to 97.96% ± 1.42% accuracy, achieving an energy efficiency of 450 Mops/s/watt.

## 1. Introduction

Monitoring wild or semi-wild animals is a useful tool for biologists and ethologists to understand their behavior, habits, possible diseases, interaction with other animals and the environment, and movement patterns. To learn and understand such details regarding wildlife, it is necessary to obtain information about movement patterns, behavior, and other biometric data such as the body temperature and heart rate of the animal during long periods of time. Thus, several studies have focused on the design and implementation of animal monitoring systems.

One of the most complex and expensive activities of animal monitoring is capturing the animal itself in order to place the tracking device onto it and ensure the device is fixed to the animal and operational for the required time. The complexity of this task requires these devices to meet a series of requirements, such as being lightweight, fault-tolerant, and low-power consuming, which make their design and implementation a technological challenge. Thus, providing biologists with a lightweight and long-battery-life device which can process a large amount of information and access it online is not an easy task.

Traditionally, animal monitoring has been a task that experts have carried out through direct observation. However, due to the presence of humans in the same territory, the observed animals do not behave as they would naturally, and data collection is limited to daytime hours [1]. In order to increase the observation time and reduce human presence, different strategies have been implemented to observe animal behavior and their interaction with the environment. One of such strategies is camera traps, which consists in the installation of cameras in the environment where the animals under study usually go to feed or rest. These cameras start recording when movement is detected on the scene and store the information in the camera. Then, the recorded video has to be recovered on site and the information is post-processed using different algorithms [2,3] to classify the animal species and obtain extra information. This strategy is minimally invasive, since it is not necessary to capture the animal in order to install any device onto it. However, it does not allow the monitoring of each animal individually.

To increase the monitoring time, it is necessary to attach a device to the animal in order to collect data that can be processed and obtain information about the animal’s behavior. This device can host some sensors, such as Global Positioning System (GPS) and Inertial Measurement Unit (IMU), among others, which generate a vast amount of raw data that can be processed by machine learning algorithms; this allows classifying some patterns which help experts to understand the behavior of the studied animal [4,5]. In the case that several animals, which can interact with each other and move in the same environment, need to be monitored, there exist other strategies based on Wireless Sensor Network (WSN). These devices can be attached to the animals and share information between them. The information is sent to the coordinator node, which transmits it to another system that is usually in the cloud [6,7]. This kind of network normally uses two types of wireless connections: one for short range, such as Bluetooth or ZigBee, and another one for large range or Internet connection, such as WiFi, GPRS, or LoRA [8]. Recent studies [9,10,11,12,13,14] have proposed collar devices consisting of a GPS module and an IMU (or an accelerometer only) for animal behavior recognition. In [9], the authors present a device which collects information from a 3-axis accelerometer and a GPS. The information obtained from 5 different behaviors (foraging, ruminating, traveling, resting, and “other”), which was stored on a card, was then used to train and evaluate a decision tree offline, achieving an 85.5% accuracy. In [10], a device, which hosts an IMU, was used to collect information and perform 5-class behavior classification of cows (grazing, walking, ruminating, resting, and “other”). A set of binary time series classifiers were trained and validated offline, achieving an 85% accuracy. In [11], they used 3-axis neck-mounter accelerometers for dairy cattle behavior classification using a decision tree. Three different behaviors were considered (eating, rumination, and lying), for which the authors reported 99.2% accuracy after training and validating the classifier in Matlab. In [12], the authors presented a system based on combining leg-mounted and collar-mounted accelerometers. The system classifies between four different behaviors (walking, feeding, lying, and standing) using a Random Forest algorithm with an 94.3% accuracy in an offline manner. In [13], the authors compared two commercial devices that provide tri-axial acceleration data and GPS information. These devices are used to obtain data related to different behaviors from giraffes, which are then used to train and evaluate a machine learning algorithm (Random Forest), achieving a mean accuracy of 88.4% over six different behaviors when using cross validation. Finally, in [14], different offline machine learning algorithms weree compared over data corresponding to four behaviors obtained from accelerometers mounted on sheep. Among the different algorithms, the best result was achieved with a Support Vector Machine (76.9% accuracy).

All these aforementioned devices store the information generated by the sensors and, after collecting it, it is processed in a local computer, where the classifier is trained and tested. In order to perform a real-time classification of the acquired data, two possible approaches could be followed. The first way consists in sending the raw information, through a radio transmitter device, to another system for post-processing; and the second one, called edge-computing, consists in processing the information in the device itself and sending the new resulting information [15,16]. The processing can be done using statistical models like [15,16] or using Embedded Neural Network (ENN) algorithms like [17]. This computing paradigm, which calls for processing the data at the edge of the device [18], has several advantages, such as real-time analysis, low latency, security, and reduced data delivery, which can be beneficial in the field of animal monitoring. One of the most expensive tasks in terms of energy consumption is the transmission of information through the radio of the monitoring device. Thus, if the rate of data to be sent is reduced, the battery life of the device is increased, which would prolong the time that the animal could be monitored without changing the battery. To reduce the sending rate, the data should be processed in the device itself. As previously commented, some devices use Artificial Neural Networks (ANNs) to process the information obtained by the sensors of the device, but this type of algorithm is power-hungry and requires exhaustive computation. To reduce the complexity of these algorithms, their parameters are quantified without significantly reducing their accuracy [19,20]. This technique allows the use of these algorithms in embedded systems using an affordable energy consumption for their purpose.

This paper presents an analysis and study of the execution of an ENN, taking as input the data generated by an IMU in different Cortex-M architectures for use in an embedded system. Different comparisons were performed between the different studied microcontrollers in terms of power consumption, execution time and performance of different ENNs implemented in them.

## 2. Materials and Methods

### 2.1. Wearable Data Acquisition Platform for Wildlife

**Figure 1:**
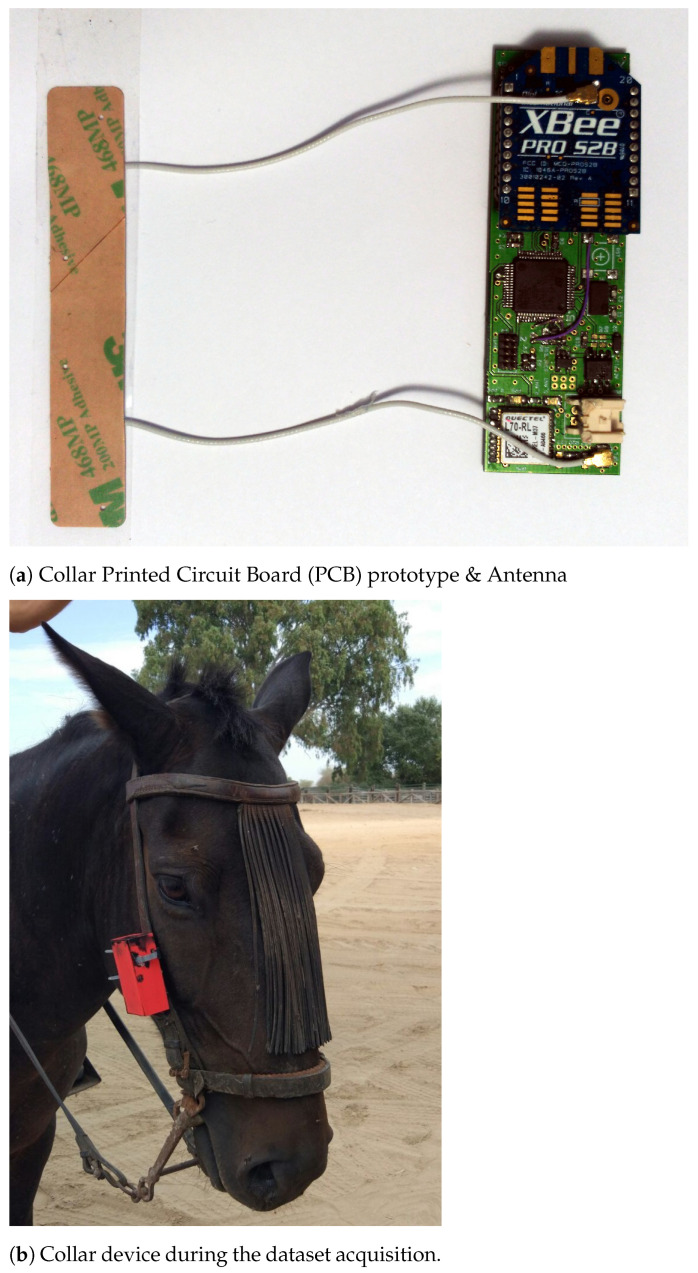
(**a**) From left to right: Antenova Asper 2.4 G/GNSS Antenna, and PCB front view, showing the microcontroller, the XBee module, and the GPS module. The IMU is under the XBee radio module, between the PCB and the module. (**b**) Horse with the collar device prototype attached.

In this work, a full custom wearable device was used and validated in real-test scenarios (Figure 1a). The aim of this device, also called collar, was to collect precise information from the animal on which it was attached by using multiple devices. A MinIMU-9V2 IMU, which consists of a LSM303DLHC 3-axis accelerometer, a L3GD20 3-axis gyroscope, and a 3-axis magnetometer, was used to measure the animal’s movement dynamics. By means of an I2C interface, it was possible to read nine independent rotation, acceleration, and magnetic measurements with 12-bit resolution for each sample and with a sample rate of 33 samples/s.

A GPS module was used along with the IMU module in order to tag each sample with the location and the time in which the sample was obtained. The periodical measures of each sensor were carried out using a low power microcontroller (STM32L152 (STMicroelectronics, Geneva, Switzerland)), which was powered using a four AAA battery pack (1.5 V, 1155 mAh each). The wearable prototype also includes an XBee module (XBee PRO S2B) for transmitting the acquired data through a wireless communication to a computer in order to be stored in a database. To ensure the correct dataset acquisition, and due to the fact that capturing a semi-wild animal is very expensive and difficult, a microSD card was added to the device in order to store the read samples and avoid data loss due to a bad wireless connection. Two different horses from completely different breeds, places were considered in this work for acquiring the data, which was done during two different seasons of the year.

### 2.2. Dataset

The IMU sensors presented in Section 2.1 were used to collect experimental data from three different horse gaits: motionless, walking, and trotting. A total of 6841 samples were obtained for the motionless behavior, 6842 for walking, and 11,512 for trotting. These data were obtained after placing the collar device in a vertical position at the right part of the horse’s head (Figure 1b), following the recommendations of experts biologists that supported us throughout this study [21].

For this study, two different datasets were considered to train and validate the Neural Network (NN). On the one hand, the first dataset consisted in the raw data obtained from the accelerometer sensor, since it was demonstrated that it is the most relevant IMU sensor for classifying between motionless, walking, and trotting [21]. On the other hand, the raw data, collected from the accelerometer, gyroscope, and magnetometer sensors, were pre-processed using a Kalman filter [22] to obtain pitch, roll, and yaw values. These pre-processed samples were used as the second dataset. In [21], the authors proved that the Kalman algorithm improves the accuracy between these three classes when training a simple NN (one hidden layer with less than 30 neurons). However, no comparison between the two kinds of data was performed for more complex networks.

### 2.3. Neural Network Models

In this work, several ANN architectures were trained and tested for each dataset (see Section 2.2). Three neurons were set for the input layer. For the hidden layers (HLs), different experiments were carried out, with models having between one and three layers, each of them with 10, 20, 30, 50, 100, 200, 300, and 500 neurons. Sigmoid activation functions and batch normalization was used after each of the aforementioned layers. Finally, a Softmax decision layer with three neurons receives the output from the last fully-connected layer and predicts the corresponding class.

K-fold stratified cross-validation was performed to measure the generalization ability of the models. This technique consisted in dividing the datasets presented in Section 2.2 into 5 sets (K=5), each of them with a proportional number of samples per class. For each fold, the networks were trained using four of the five sets (80% of the dataset) for 50,000 epochs, and were validated using the remaining set (20% of the dataset). This way, for each experiment, the networks were trained and validated a total of five times with different data. Therefore, summing up all the different possible combinations, the number of models that were trained and tested were: 2 datasets × 3 HLs × 8 different numbers of neurons in the HL× 5 cross-validation sets, which produced a total of 240 models. All of these models were trained and tested using TensorFlow (https://www.tensorflow.org, accessed on 23 April 2021) [23] and Keras (https://keras.io, accessed on 23 April 2021) [24]. Adam was used as optimizer, with a learning rate of 0.01 and a batch size of 64 samples. The class imbalance was taken into account using Keras’ *class_weight* parameter, which automatically weighs each class based on the number of training samples used. The final results for a specific experiment were presented as the mean accuracy and their standard deviation calculated over the five cross-validation folds.

### 2.4. Full Integer Quantization

TensorFlow Lite (https://www.tensorflow.org/lite, accessed on 23 April 2021) is an open-source deep learning framework for on-device inference using pre-trained models. It provides different tools for converting and running TensorFlow and Keras models on embedded devices and microcontrollers. Some of these tools are dedicated to the quantization of pre-trained models by reducing the precision of the weights of the NN and its operations, leading to better performance and lighter model size, sacrificing a small loss of precision and accuracy of the classification.

Different post-training quantization techniques are already implemented in TensorFlow Lite, such as dynamic range quantization, float16 quantization, and full integer quantization (FIQ) (https://www.tensorflow.org/lite/performance/post_training_quantization, accessed on 23 April 2021) [25]. In this work, FIQ is studied, since it is optimized for deploying models on microcontrollers. This method consists in reducing all model math to 8-bit integer values, reducing the peak memory usage and improving the latency of the classification on the microcontroller. Following the documentation provided in TensorFlow Lite, this method achieves 4× smaller models with 2×−3× speedup.

### 2.5. Integrated Development Environment

STM32CubeIDE (https://www.st.com/en/development-tools/stm32cubeide.html, accessed on 23 April 2021) is a multi-OS Integrated Development Environment (IDE) for C/C++ development, with peripheral configuration, code generation, code compilation, and debug features for STM32 microcontrollers. Hundreds of plugins can be integrated within STM32CubeIDE in order to extend the IDE’s functionalities, including STM32CubeMX, which is a standalone GUI-based software tool for initializing and configuring a project for a specific microcontroller and platform.

Within this set of tools, STM32Cube.AI (https://www.st.com/content/st_com/en/stm32-ann.html, accessed on 23 April 2021) can be found. STM32Cube.AI is an extension pack that is fully integrated into STM32CubeMX and allows mapping and running pre-trained ANNs on the broad STM32 microcontroller family. It allows for a fast and automatic conversion of pre-trained ANNs into optimized code that can run on a microcontroller. This tool also reports feedback on the performance of the converted NN in the microcontroller, comparing it with the original model in terms of accuracy, error, and other useful metrics.

### 2.6. Cortex-M Microcontrollers

To evaluate the ENN performance from different points of view, two 32-bit Cortex-M processors were used. Firstly, the NN was deployed in a low-power Cortex-M4 processor, specifically a STM32L475 (https://www.st.com/en/microcontrollers-microprocessors/stm32l475vg.html, accessed on 23 April 2021), which works at 80 MHz. Then, an advanced Cortex-M7 (STM32F746 (https://www.st.com/content/st_com/en/products/microcontrollers-microprocessors/stm32-32-bit-arm-cortex-mcus/stm32-high-performance-mcus/stm32f7-series/stm32f7x6/stm32f746vg.html, accessed on 23 April 2021)) with a clock of 216 MHz and a superscalar architecture was used. STM32L475 and STM32F476 have 1 Mbytes of flash memory, and 128 and 320 Kbytes of RAM memory, respectively. These data are summarized in Table 1. The aim of choosing both microcontrollers is to offer a criterion in terms of power consumption and execution time for the same ANNs between the two architectures.

Since the executed code in both microcontrollers is the same, there is no difference in the NN complexity, accuracy, or even in the confusion matrix. Therefore, with similar inputs, the same output is obtained. However, there is a considerable difference in terms of power consumption and execution time.

## 3. Results

### 3.1. Cross-Validation Results

**Figure 2:**
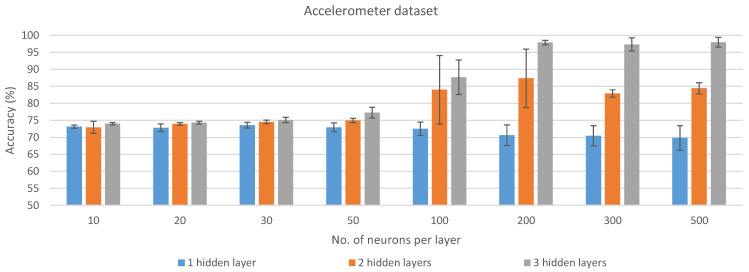
Average of the results obtained with the accelerometer dataset over the different cross-validation folds for each of the analyzed configurations.

**Figure 3:**
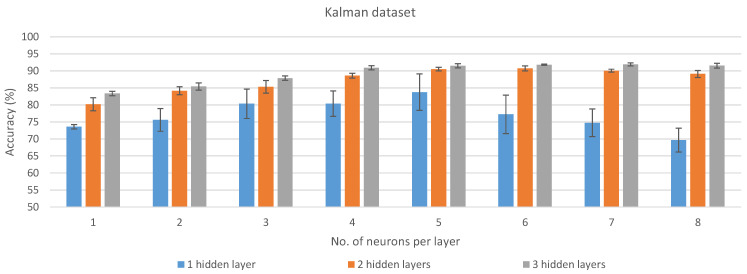
Average of the results obtained with the Kalman dataset over the different cross-validation folds for each of the analyzed configurations.

The results obtained from each ANN model for the two datasets after training and validating them using TensorFlow and Keras are presented in Figure 2 and Figure 3. These results are the average of the 5 cross-validation folds, with their corresponding standard deviation. The values from which the figure was generated are also presented in Table A1. The results for each cross-validation fold for all the experiments that were carried out are detailed in Table A2. The aforementioned table also includes the mean precision, recall, F1-score, and balanced accuracy per fold. As can be seen, for the classification of the three studied horse gaits, the accelerometer data are more representative than the data obtained after processing the output of the IMU with the Kalman filter. However, to achieve better results with the accelerometer data, a higher number of HLs in the model and neurons per HL have to be used, which directly affects the power consumption of the hardware platform in which the model is deployed, as well as the processing time needed for every input sample.

For each dataset and number of HLs, the best models in terms of average accuracy across the number of neurons in the HL were selected for further implementation and analysis in different hardware platforms (see Section 2.6). These models are highlighted in bold in Table A1 and correspond to 1 HL with 30 neurons (73.57% ± 0.83% accuracy), 2 HLs with 200 neurons each (87.36% ± 8.57% accuracy), and 3 HLs with 500 neurons each (97.96% ± 1.42% accuracy) for the accelerometer dataset. For the Kalman dataset, the models selected are: 1 HL with 100 neurons (83.76% ± 5.37% accuracy), 2 HLs with 200 neurons each (90.73% ± 0.71% accuracy), and 3 HLs with 300 neurons each (91.92% ± 0.45% accuracy).

The already-trained selected models obtained with Keras (.h5) were then converted to TensorFlow Lite (.tflite) models in order to integrate them in the different microcontrollers and hardware platforms presented in Section 2.6. Moreover, a full integer quantization was also performed to the converted models, which, as presented in Section 2.4, allows achieving smaller and faster versions of these in terms of memory size and performance, making them more appropriate for being deployed in a microcontroller.

### 3.2. Hardware Integration Results

The selected models mentioned in the previous Section 3.1 were deployed on two different platforms (Section 2.6) using STM32Cube.AI (Section 2.5). Different metrics were considered in order to compare original models trained in TensorFlow and Keras with the TensorFlow Lite and full integer quantized ones deployed on the microcontrollers. Two of the selected models could not be deployed on the different hardware platforms due to their limitations on Flash memory. These correspond to TensorFlow Lite versions of the selected models with 3 HLs.

**Figure 4:**
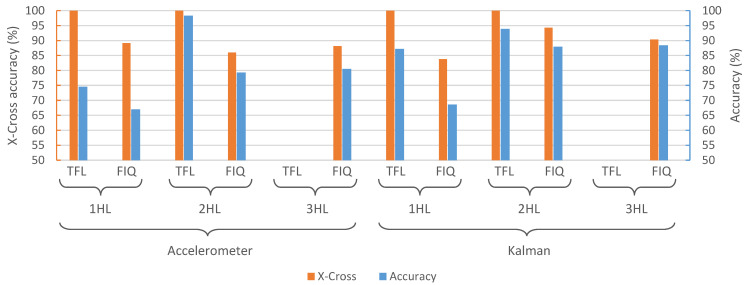
X-cross accuracy and accuracy of the different ANN models deployed on the different hardware platforms presented in Section 2.6. Since these results are hardware-independent, no distinction between them is made. TFL stands for TensorFlow Lite, while FIQ refers to Full Integer Quantization.

Figure 4 presents the X-cross accuracy and the accuracy obtained from the different selected models after deploying them in the two microcontrollers considered in this work. These two parameters represent the difference in terms of accuracy with the model trained in TensorFlow and Keras, that is, before converting them to the TensorFlow Lite format and quantizing them, as well as implementing them on the hardware platforms. Therefore, an X-cross accuracy of 100% means that the model has the same accuracy as the original model. As was previously described, both microcontrollers provide the same X-cross accuracy, since the code executed is the same. Therefore, these results are reported for the deployed ANN, and not for its execution on a specific processor. Figure 4 shows that all the different TensorFlow Lite models achieve the same results as their software counterparts. However, when performing the full integer quantization, the X-cross accuracy is lower, and thus the accuracy decreases. According to the data presented in Figure 4, X-cross accuracy is 100% in TFL conversions, whereas in FIQ conversions, this value ranges between 94% and 84%. As a benefit of FIQ ANNs having lower accuracy, they have better memory requirements. The improvement in execution time depends on network complexity, as we discuss next. According to specific applications, a balance between TFL and FIQ has to be achieved when looking for better performance in terms of classification, execution time, resources, and power demand.

**Figure 5:**
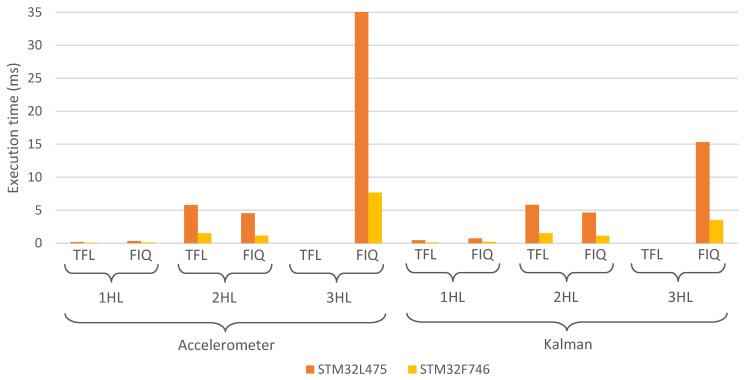
Execution time required for the ANN to perform a prediction for each of the two hardware platforms presented in Section 2.6.

Figure 5 shows the execution time for each of the proposed models, comparing between the two hardware platforms in which they were deployed. From the reported results, it can clearly be seen that the quantized model allows a faster execution of the ANN, since the complexity of the model was reduced in this process. As expected, the greater the number of HLs in the ANN, the longer the time it requires to perform a prediction. On average, the STM32F746 microcontroller needs 3.6 times less execution time to perform a classification with respect to STM32L475. If we compare the execution time between TFL models with FIQ models, different results can be found depending on the ANN size in both microcontrollers. For 1 HL networks, FIQ models are 70% slower than TFL models, but for 2 HL, FIQ models are 30% faster than TFL models.

**Figure 6:**
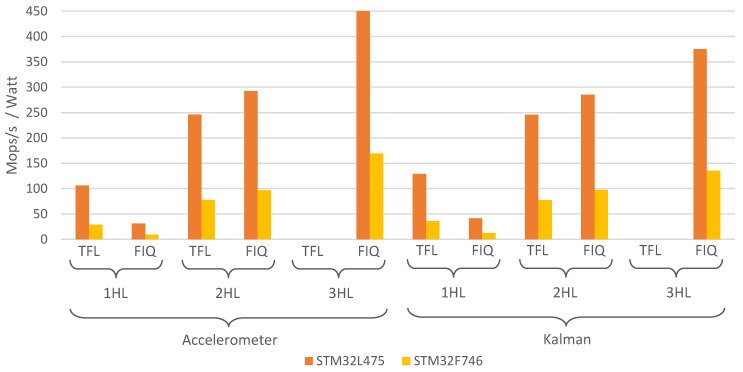
Mops/s per Watt for each of the studied models and hardware platforms.

Figure 6 compares the different models considered in this approach in terms of Mops/s/Watt ($10^{6}$ operations per second, per watt). This value is widely used when comparing the performance of embedded devices and hardware platforms. The plot shows that, in terms of performance, STM32L475 achieves better results (more than 2 times better than those of STM32F746), being able to execute more operations per demanded watt. Again, differences between 1 HL and 2 HL models can be found when comparing TFL and FIQ. For 1 HL, TFL models are 75% more efficient than FIQ models, whereas for 2 HL models, FIQ are 25% more efficient than TFL.

**Figure 7:**
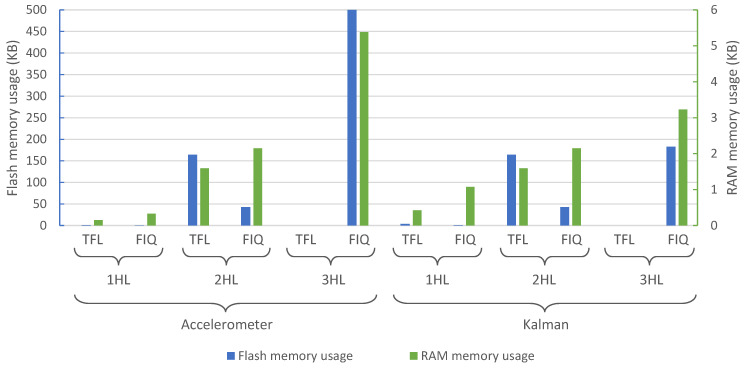
Flash memory usage for each of the studied models and hardware platforms.

Figure 7 shows the memory usage in terms of flash and RAM memories, which are the same for both microcontrollers. Regarding the flash memory demand, TFL models need, on average, 3.4 times more flash memory than FIQ. This is due to the fact that TFL models store the ANN weights as 4-byte floating point numbers, while FIQ uses 1 byte per weight, and some overhead is added due to the network structure, which must be data-representation independent. Regarding RAM memory, FIQ demands 1.8 times more memory than TFL on average. This disparity could be related to the internal memory organization and data packaging.

Finally, the battery consumption was estimated for each microcontroller in order to estimate battery life. The results obtained are presented in Table 2. The battery considered in this study is a LiSOCL2, with a capacity of 3400 mAh. It was suggested by STM32CubeMX taking the smallest compatible battery based on the maximum continuous current demanded by the microcontroller. This battery has a weight of 50 grams and, therefore, it is suitable for being placed on horses. Our experiment consisted in the estimation of battery life for the best case (1 HL NN), midpoint case (2 HL NN) and worst case (3 HL NN) when considering an execution rate of 10 predictions per second. With low complex NNs, the STM32L475 can be powered for more than 1 year, but, in the case of the STM32F746, this number is reduced to 3 months. With medium complexity NNs, this estimations decays approximately to 65% and 35% of the original time, respectively. Finally, for higher complexity networks, the estimated battery life is 3 months for the STM32L475 microcontroller, and 15 days for the STM32F746.

Table A3 contains all the numerical results used to obtain the figures that are shown in this section.

## 4. Discussion

When comparing Kalman and raw-accelerometer datasets, the former achieves better results in lower complex networks (smaller number of HLs and neurons). On the other hand, if we look for the best results in terms of accuracy, these are obtained with the accelerometer data when using a NN with a greater number of layers. Even though having less neurons and layers could also mean having a lower power consumption and a longer battery life, the power consumption of calculating the Kalman filter per input data was not considered in this work, since we only focused on the NN.

Regarding the different hardware platforms considered in this study, the Cortex-M7 takes 3.6 times less execution time to perform a prediction. On the other hand, the low power Cortex-M4 architecture needs half of the power consumption to perform the same classification, with a battery life six times bigger on average. Making a decision between them strongly depends on the target application. In our specific case, since the system is designed for wildlife monitoring, the low-power solution of the STM32L475 is more suitable for this kind of application, which is also applicable to general wearable devices.

An interesting comparison can also be found between TFL and FIQ models. Generally, TFL models achieve better accuracies, although with larger networks. Contrary to TFL, FIQ models demand less resources and are faster. As a counterpart, FIQ models are less efficient in terms of energy consumption per prediction. Summarizing, in the case of wildlife monitoring, choosing between TFL and FIQ models is not clear. Researchers should look for the balance between accuracy, battery life, and microcontroller’s capabilities in order to find the most appropriate approach depending on the task.

In this work we analyzed and tested a prototype particularly developed for semi-wild horses, since it is a simpler and more convenient case study than others that include smaller and wilder animals in terms of capturing them and inducing them to perform a set of gaits. Testing the prototype system on horses was the recommended scenario and approach by the expert biologists that supported this project. If this approach is to be used for other semi-wild or wild animals, their habits should be studied and those of interest should be recorded and used to train a neural network in a supervised manner. A recent study [26] proposed a novel approach for using data from captive individuals to infer wildlife behavior, which could be taken as an inspiration for future works.

In previous state-of-the-art solutions, such as [9,10,11,12,13,14], authors evaluated the classifier in a local computer without providing on-device or cloud-based real-time animal behavior classification. This fact restricts their implementation in a real scenario, since predicting behavioral patterns of the animals after removing the monitoring device from them and processing the stored information is not the optimal use case. Our proposed system is capable of performing real-time online classification, allowing expert biologists to access the data as soon as it is predicted by the collar devices. In terms of battery life and power consumption, the aforementioned studies report a battery life of 12–14 days [9], 14 days [10], 7 days [12], and 17 days [13], respectively. When using the lower power consumption microcontroller from the two options that were considered in this work, we obtain an estimated battery life of 88 days in the worst case, which is 5 times more battery life than [13], taking into consideration that, in our work, we also include the processing of the data and the prediction from the ENN in this estimation. However, if a midpoint case is considered, the estimated battery life is more than 15 times higher than the one from [13].

Even though the proposed system was evaluated using a 5-fold cross-validation scheme, which demonstrates a good generalization of the model, using only two horses could be seen as a limitation of this study. Future works will consider gathering a bigger dataset with different horses in order to have more variation in the obtained data.

## 5. Conclusions

In this work, different NNs for classifying between three different horse gait patterns were trained and tested using two different datasets (Kalman and raw accelerometer data) obtained from an IMU sensor placed on horses. A total of 24 different ANN models per dataset were trained using a 5-fold cross-validation scheme in order to compare them in terms of complexity and accuracy. The best models were then converted to microcontroller-friendly TensorFlow Lite (TFL) models and quantized (FIQ) in order to reduce the complexity of the network. These two versions of the aforementioned models were deployed on two different microcontrollers (STM32L475 and STM32F746) for further analysis in terms of performance, power consumption, energy efficiency, and battery life. The proposed system improves the battery life of current state-of-the-art solutions by more than five times in the worst case scenario. Furthermore, recent works present animal behavior classification systems where the inference is performed outside of the collar device, either by sending the raw sensor information to a computer or by recovering the SD card from the collar after some days. On the other hand, the edge-computing system presented in this work is able to perform on-device real-time animal behavior classification, reducing data transmissions and, thus, reducing power consumption, together with providing biologists access to the data anytime.

The results show that raw accelerometer data achieve better accuracy results for larger ANNs (around 98% accuracy for three different horse gaits). When embedded on the microcontroller, FIQ models obtain lower classification results and higher RAM memory usage, while benefiting from lower prediction time and flash memory usage. The comparison between the two microcontrollers and the different parameters considered in this study was analyzed. The choice of which microcontroller, model, number of layers, neurons per layer, and dataset to use depends on the particular application, and researchers must select the best one according to their restrictions (e.g., maximizing either battery life or accuracy).

## Figures and Tables

**Table 1:** Main features of STM32L475 and STM32F476 microcontrollers.

Device	Architecture	Features	Clock (MHz)	RAM (kBytes)	Flash (Mbytes)	Current (mA)
STM32L475	Cortex-M4	Ultra-low-power Single precision FPU	80	128	1	10.7
STM32F746	Corterx-M7	High-performance DSP with FPU	216	320	1	130.1

**Table 2:** Estimated battery life for a Li-SOCL2(A3400) battery when performing 10 classifications per second with the studied microcontrollers and FIQ network architectures.

Device	Best Case (1 HL)	Midpoint Case (2 HL)	Worst Case (3 HL)
STM32L475	404 days	263 days	88 days
STM32F746	101 days	35 days	15 days

## Abbreviations

The following abbreviations are used in this manuscript:

ANN	Artificial Neural Network
ENN	Embedded Neural Network
FIQ	Full Integer Quantization
GPS	Global Positioning System
HL	Hidden Layer
IDE	Integrated Development Environment
IMU	Inertial Measurement Unit
NN	Neural Network
TFL	TensorFlow Lite
WSN	Wireless Sensor Network

## Appendix A

**Table A1:** Average of the results obtained over the different cross-validation folds for each of the studied configurations. The best results for each dataset and number of layers are highlighted in bold.

	Dataset	No. HL	No. Neurons							
			10	20	30	50	100	200	300	500
Model	Accel	1	73.13 ± 0.48	72.81 ± 1.11	**73.57 ± 0.83**	72.96 ± 1.29	72.49 ± 1.97	70.61 ± 3.02	70.43 ± 2.96	69.82 ± 3.61
		2	72.93 ± 1.77	73.98 ± 0.37	74.53 ± 0.54	74.96 ± 0.61	84.01 ± 10.06	**87.36 ± 8.57**	82.89 ± 1.07	84.43 ± 1.64
		3	73.99 ± 0.35	74.32 ± 0.41	75.07 ± 0.79	77.24 ± 1.59	87.67 ± 5.10	97.88 ± 0.63	97.34 ± 1.92	**97.96 ± 1.42**
	Kalman	1	73.59 ± 0.65	75.60 ± 3.34	80.35 ± 4.32	80.37 ± 3.73	**83.76 ± 5.37**	77.24 ± 5.65	74.76 ± 4.05	69.64 ± 3.50
		2	80.21 ± 1.92	84.18 ± 1.18	85.31 ± 1.84	88.60 ± 0.68	90.53 ± 0.50	**90.73 ± 0.71**	90.07 ± 0.44	89.11 ± 1.02
		3	83.36 ± 0.66	85.40 ± 1.05	87.88 ± 0.64	90.91 ± 0.63	91.51 ± 0.60	91.81 ± 0.18	**91.92 ± 0.45**	91.55 ± 0.69

**Table A2:** Results obtained for the different cross-validation folds for each of the studied configurations.

			Accuracy per Fold (%)					Metrics (Mean) (%)				
Data	HL	Neurons	1	2	3	4	5	Accuracy	Precision	Recall	F1-Score	Balanced Accuracy
**Accel.**	**1**	10	72.52	73.40	73.15	73.88	72.74	73.14	73.36	73.14	73.05	75.24
		20	72.08	73.91	70.98	73.68	73.43	72.82	72.98	72.82	72.69	74.96
		30	72.02	73.95	74.53	73.61	73.77	73.58	73.75	73.58	73.48	75.63
		50	73.46	74.00	72.99	73.88	70.48	72.96	73.14	72.96	72.86	75.02
		100	71.76	68.98	74.23	74.23	73.27	72.49	72.68	72.49	72.31	74.73
		200	66.81	74.20	74.18	69.08	68.78	70.61	70.81	70.61	70.25	73.25
		300	67.14	74.20	68.52	73.82	68.51	70.44	70.83	70.44	70.11	73.11
		500	66.76	74.43	74.07	67.03	66.85	69.83	70.05	69.83	69.45	72.55
	**2**	10	73.28	74.51	74.25	73.08	69.56	72.94	73.30	72.94	72.87	75.03
		20	74.31	74.34	74.20	73.61	73.45	73.98	74.28	73.98	73.96	75.90
		30	74.39	75.03	75.13	73.61	74.53	74.54	74.83	74.54	74.52	76.41
		50	74.89	75.01	75.93	73.98	75.02	74.97	75.20	74.97	74.93	76.81
		100	96.18	76.48	76.64	74.35	96.43	84.01	84.05	84.01	83.99	85.02
		200	80.28	98.34	80.42	80.42	97.38	87.37	87.37	87.37	87.35	88.11
		300	83.08	83.71	82.45	81.08	84.17	82.90	82.91	82.90	82.88	83.76
		500	82.32	87.02	83.78	83.55	85.51	84.44	84.46	84.44	84.43	85.51
	**3**	10	73.51	74.39	74.12	73.65	74.30	73.99	74.34	73.99	73.97	75.94
		20	74.41	74.99	74.20	73.70	74.31	74.32	74.65	74.32	74.30	76.24
		30	74.66	76.12	75.65	73.84	75.11	75.08	75.37	75.08	75.05	76.91
		50	77.75	78.03	78.23	74.07	78.12	77.24	77.43	77.24	77.22	78.79
		100	97.42	82.91	84.37	87.02	86.66	87.68	87.72	87.68	87.67	88.35
		200	98.60	96.92	97.88	98.53	97.51	97.89	97.89	97.89	97.89	98.03
		300	98.53	93.67	97.36	99.15	98.00	97.34	97.35	97.34	97.35	97.55
		500	95.14	98.87	98.74	98.82	98.23	97.96	97.97	97.96	97.96	98.11
**Kalman**	**1**	10	74.57	73.56	73.79	73.54	72.51	73.59	73.53	73.59	73.08	74.77
		20	77.74	76.44	75.17	79.20	69.47	75.60	75.46	75.60	75.15	76.85
		30	83.4	75.98	83.18	84.88	74.31	80.35	80.24	80.35	80.15	81.61
		50	73.70	80.70	80.69	85.23	81.55	80.38	80.29	80.38	80.16	81.52
		100	88.22	88.40	74.97	87.21	80.03	83.77	83.76	83.77	83.60	84.82
		200	76.20	88.27	75.29	73.84	72.60	77.24	77.23	77.24	76.80	78.38
		300	72.82	80.16	70.75	79.11	70.99	74.76	74.90	74.76	74.25	75.77
		500	73.30	70.32	63.07	69.81	71.73	69.65	70.30	69.65	68.26	71.16
	**2**	10	81.47	79.45	81.50	76.76	81.90	80.22	80.11	80.22	80.06	81.50
		20	84.83	85.44	82.56	82.95	85.14	84.18	84.12	84.18	84.10	85.34
		30	82.88	88.06	83.76	86.40	85.48	85.32	85.28	85.32	85.23	86.37
		50	89.35	87.97	88.50	87.76	89.42	88.60	88.60	88.60	88.56	89.43
		100	90.19	91.51	90.43	90.47	90.09	90.54	90.53	90.54	90.50	91.28
		200	91.14	90.98	90.66	89.41	91.49	90.74	90.72	90.74	90.70	91.47
		300	90.26	89.37	89.97	90.73	90.02	90.07	90.07	90.07	90.03	90.83
		500	88.26	89.76	87.57	89.65	90.31	89.11	89.09	89.11	89.06	89.89
	**3**	10	84.08	83.85	83.07	83.60	82.22	83.37	83.34	83.37	83.27	84.53
		20	84.54	87.07	84.05	85.87	85.48	85.40	85.40	85.40	85.32	86.55
		30	87.25	87.94	87.23	87.99	89.00	87.88	87.89	87.88	87.82	88.84
		50	91.83	91.10	90.08	91.26	90.32	90.92	90.92	90.92	90.88	91.70
		100	91.71	91.19	90.71	91.42	92.53	91.51	91.52	91.51	91.48	92.20
		200	91.53	91.90	91.76	92.09	91.81	91.82	91.83	91.82	91.79	92.50
		300	92.57	91.19	92.16	91.79	91.90	91.92	91.93	91.92	91.90	92.56
		500	90.93	91.37	92.24	90.73	92.48	91.55	91.54	91.55	91.52	92.26

**Table A3:** Results obtained for each of the implemented models on the two microcontrollers considered. The complexity of the models in terms of number of operations is also presented.

Microcontroller	Clock Freq. (MHz)	Data	HL	Model	Flash (KB)	RAM (KB)	Complexity (ops)	Current (mA)	Power (mW)	Energy (mJ)	Processing time (ms)	CPU cycles	cycles/MAC	Acc (%)	l2r	X-Cross acc (%)	Mops/s/Watt
STM32L475	80	Accel	1	TFLite	1.14	0.152344	630	10.7	32.1	5.9385	0.185	14794	23.48	74.53	0.732473	100	106.087
				Quant.	0.402344	0.330078	351	10.7	32.1	11.2671	0.351	28084	80.01	67.00	0.808616	89.19	31.152
			2	TFLite	164.14	1.59	45690	10.7	32.1	185.7627	5.787	462965	10.13	98.34	0.172436	100	245.958
				Quant.	42.62	2.15	42481	10.7	32.1	145.2525	4.525	361988	8.52	79.25	0.624254	86.03	292.463
			3	TFLite	-	-	-	-	-	-	-	-	-	-	-	-	-
				Quant.	500.04	5.38	507591	10.7	32.1	1125.8112	35.072	2805759	5.53	80.47	0.60562	88.15	450.858
		Kalman	1	TFLite	3.6	0.425781	1890	10.7	32.1	14.6376	0.456	36442	19.28	87.21	0.478239	100	129.119
				Quant.	1.21	1.08	981	10.7	32.1	23.5293	0.733	58680	59.82	68.57	0.771917	83.82	41.692
			2	TFLite	164.14	1.59	45690	10.7	32.1	186.0195	5.795	463579	10.15	93.86	0.316368	100	245.619
				Quant.	42.62	2.15	42481	10.7	32.1	148.944	4.64	371176	8.74	87.88	0.462954	94.27	285.214
			3	TFLite	-	-	-	-	-	-	-	-	-	-	-	-	-
				Quant.	182.86	3.23	184581	10.7	32.1	491.9646	15.326	1226069	6.64	88.40	0.439292	90.34	375.191
STM32F746	216	Accel	1	TFLite	1.14	0.153846	630	130.1	390.3	21.8568	0.056	12133	19.26	74.53	0.734273	100	28.823
				Quant.	0.402344	0.330078	351	130.1	390.3	38.2494	0.098	21115	60.16	67.00	0.808616	89.19	9.176
			2	TFLite	164.14	1.59	45690	130.1	390.3	588.5724	1.508	325814	7.13	98.34	0.172436	100	77.628
				Quant.	42.62	2.15	42481	130.1	390.3	439.0875	1.125	242919	5.72	79.25	0.624254	86.03	96.748
			3	TFLite	-	-	-	-	-	-	-	-	-	-	-	-	-
				Quant.	500.04	5.38	507581	130.1	390.3	2998.6749	7.683	1659565	3.27	80.47	0.60562	88.15	169.268
		Kalman	1	TFLite	3.6	0.425781	1890	130.1	390.3	52.6905	0.135	29129	15.41	87.21	0.478239	100	35.869
				Quant.	1.21	1.08	981	130.1	390.3	80.7921	0.207	44656	45.52	68.57	0.771917	83.82	12.142
			2	TFLite	164.14	1.59	45690	130.1	390.3	590.5239	1.513	326834	7.15	93.86	0.316368	100	77.371
				Quant.	42.62	2.15	42481	130.1	390.3	435.5748	1.116	241128	5.68	87.88	0.462954	94.27	97.528
			3	TFLite	-	-	-	-	-	-	-	-	-	-	-	-	-
				Quant.	182.86	3.23	184581	130.1	390.3	1360.5858	3.486	753058	4.08	88.4	0.439292	90.34	135.662
